# Detection and quantification of *Anopheles gambiae *sensu lato mosquito larvae in experimental aquatic habitats using environmental DNA (eDNA).

**DOI:** 10.12688/wellcomeopenres.14193.1

**Published:** 2018-03-08

**Authors:** Joel Odero, Bruno Gomes, Ulrike Fillinger, David Weetman

**Affiliations:** 1 Malaria Programme, International Centre of Insect Physiology and Ecology, Thomas Odhiambo Campus, P.O. Box 30, Mbita, 40305, Kenya; 2 Department of Vector Biology, Liverpool School of Tropical Medicine, Liverpool, L3 5QA, UK

**Keywords:** Environmental DNA (eDNA); An. gambiae s.s.; An. arabiensis; larvae; aquatic habitats; monitoring.

## Abstract

**Background:** Growing insecticide resistance and changes in biting and resting behavior of malaria vectors threaten efficacy of insecticide treated nets and indoor residual spraying. Larval source management (LSM) is a promising approach that can target mosquitoes irrespective of their behavior as adults. However, the use of traditional monitoring methods for immature stages of *Anopheles* mosquitoes is a major challenge to LSM due to the variability in their breeding habitats.  We evaluate the use of an environmental DNA (eDNA) analysis technique in monitoring *Anopheles gambiae *sensu lato larvae in experimental aquatic habitats.

**Methods:** eDNA was simultaneously sampled and extracted from different volumes of water, number of larvae, and occupation time. Larval presence was detected using PCR and eDNA concentration in samples from 1 L habitats quantified using an *IGS* and *cyt b* TaqMan assays. The limit of detection of the two assays was tested and larval density correlated with eDNA positivity.

**Results:** 74% of replicates in the 50 mL habitats were PCR positive with at least 6h required to get a signal from a single larva (0.02 larvae/mL). All 12 replicates where 1 L of water was used were positive with stronger PCR bands than replicates with the same larval density in 50 mL for 24 h. There was a correlation between larval densities and eDNA detection in both assays: *IGS*, *r* = 0.503, p = 0.047; and *cyt b,* *r* = 0.558, p = 0.025. There was stochasticity in eDNA detection rates, using both PCR and qPCR across all the dilutions.

**Conclusion:** This study has demonstrated the potential use of eDNA analysis for detection and quantification of *An. gambiae *s.s. mosquito larvae in aquatic habitats. The stochasticity observed in eDNA detection suggest that this technique is best for monitoring aquatic habitats with many larvae at low densities.

## Introduction

Malaria remains actively transmitted in about 55 countries in sub-Saharan Africa ( World Health Organization, 2017). An upscale in distribution and usage of vector control interventions combined with prompt case detection and treatment has seen malaria cases drop significantly over the past decade, with vector control accounting for almost 80% of this reduction ( Bhatt *et al.*, 2015). However, growing insecticide resistance ( Ranson & Lissenden, 2016) and behaviors such as outdoor biting and resting ( Gatton *et al.*, 2013; Killeen, 2014) threaten the efficacy of insecticide treated nets and indoor residual spraying necessitating the requirement of additional vector control tools ( Hemingway, 2014), preferably targeting different parts of the mosquito life cycle ( Killeen, 2014).

Larval source management (LSM) is a promising approach ( Tusting *et al.*, 2013) that can target mosquitoes irrespective of their behavior as adults, and with a greater range of pesticides available, can potentially overcome problems of adult insecticide resistance ( Fillinger & Lindsay, 2011). LSM has historically been employed to eliminate malaria in many countries across the globe ( Killeen *et al.*, 2002) However, the uptake of this intervention is impeded by the lack of knowledge of aquatic habitats that are the most preferred; if better understood, this would permit a spatially targeted intervention of the larvae sites ( Gu *et al.*, 2008). The success of an LSM strategy requires accurate and replicable methods for monitoring the aquatic stages of disease vectors.

Monitoring the immature stages of *Anopheles* is traditionally done by sampling eggs, larvae, or pupae ( Silver, 2008). This can be challenging due to the highly variable nature of the breeding sites ranging from tiny, easily-sampled pools (e.g. hoof prints and puddles) to large and complex water bodies such as river or lake margins, and rice fields ( Minakawa *et al.*, 2004), which can lead to sampling biases and under/overestimation of larval densities in different habitats ( Silver, 2008). Despite broad-scale ecological niche differences the most important malaria vectors within the *Anopheles gambiae* s.l. species complex frequently share larval habitats and are morphologically indistinguishable ( Chen *et al.*, 2008), which means that traditional ecological sampling approaches must be supplemented by DNA-based analyses to identify and quantify the different species in a given habitat.

Environmental DNA (eDNA) analysis is an increasingly popular method used for ecological surveillance of both aquatic and non-aquatic habitats that can detect the presence of organisms, and potentially estimate density without direct physical sampling of the organisms ( Thomsen & Willerslev, 2015). eDNA is the residual DNA shed by all aquatic organisms in the form of faecal waste, urine, dead skin, gametes or via post-mortem degradation. This residual DNA can be detected by molecular techniques allowing inference of the presence of the organisms from habitat samples ( Ficetola *et al.*, 2008). In natural habitats, eDNA concentration is affected by several factors such as: the density of the target species, temperature, microbial activity in the habitat, DNA depurination (nucleic acid degradation) and exposure to ultraviolet light ( Barnes *et al.*, 2014; Pilliod *et al.*, 2014). eDNA concentration under experimental sunny conditions has been shown to reduce by 80% after just one day and up to 98% in two days ( Pilliod *et al.*, 2014). Hence most DNA detection is expected to indicate a current or recent colonization of the habitat ( Piaggio *et al.*, 2014), making it a suitable method for contemporary surveillance of aquatic populations. eDNA analysis has been used for detection of a variety of aquatic animals and, where compared usually correlates well with presences/absence quantification using conventional sampling methods ( Dejean *et al.*, 2011; Minamoto *et al.*, 2012; Spear *et al.*, 2015).

Only one aquatic survey with eDNA analysis has been performed in mosquitoes to date in which three *Aedes* species that are invasive in Europe *(Aedes albopictus*, *Aedes j. japonicus*, and *Aedes koreicus*) ** were monitored in small human container habitats: typical breeding sites for *Aedes* spp. ( Schneider *et al.*, 2016). The highest probability of larval detection came from eDNA analysis using quantitative PCR (96%), with both a more complex shotgun sequencing-based method and traditional larval sampling surveys showing lower sensitivity (86% and 89%, respectively), confirming the efficiency of the relatively simple and inexpensive qPCR technique.

The current study is a pilot work conducted under controlled laboratory conditions that applies eDNA analysis in *Anopheles* species for the first time. We evaluated the detection and relative quantification of eDNA of *Anopheles* larvae using two qPCR assays, one of which was also capable of simultaneous differentiation of the two key East African malaria vectors *An. gambiae s.s.* and *An. arabiensis.* This latter method appears very promising for application in field eDNA studies, and we highlight the strengths and limitations of the technique.

## Methods

### Initial eDNA PCR primer design and validation

We designed eDNA PCR primers as an initial step to test performance of ChargeSwitch® Forensic DNA Purification Kit, Invitrogen in eDNA isolation and to ensure that the biological negative controls gave negative results. The primers were designed from the *An. gambiae* s.l. *cytochrome b* gene ( *cyt b*) obtained from the full mitochondrial DNA (mtDNA) sequence of *An. gambiae* s.s. (VectorBase; L20934.1). The region 10413–11549 was screened against a nonredundant database using NCBIs Primer-BLAST tool ( Ye *et al.*, 2012) restricting the product size for 70–120bp, to provide product sizes suitable for efficient qPCR amplification of degraded DNA ( Thomsen & Willerslev, 2015). Two primer pairs; 1) Forward 5’ TCCTAGCTATACACTATGCCGC3’, Reverse 5’ ATTTGTCACGCTAACGGAGCT3’ and 2) Forward 5’AGCTATACACTATGCCGCAGAT3’, Reverse 5’AAGCTCCGTTAGCGTGACAAA3’ were validated by PCR with separate serial dilutions of 1:10 and 1:100 of *An. gambiae* s.s. control gDNA.

Each reaction consisted of 17.8 μL PCR water, 2.5 μL 10X Dream Taq Green Buffer, 0.5 μL of 10 mM dNTP mix, 0.2 μL Dream Taq DNA Polymerase, 0.5 μL of each primer pair and 3 μL of DNA template. The thermocycler conditions were 1 cycle 95°C for 5 minutes followed by 35 cycles of 95°C for 1 minute, 60°C for 1 minute, 72 °C for 1 min and a final extension at 72 °C for 5 minutes and held at 10°C. PCR products were cleaned using ExoSAP-IT ^®^ (Affymetrix, UK) and sequenced commercially (SourceBioscience, UK) to ensure that they only amplified *An. gambiae* s.l. ** *cyt b* gene. Sequences then aligned against reference *An. gambiae* mitochondrial *cyt b* sequence using CodonCode Aligner software (Version 4.2.7). Primer pair 1 showed consistent amplification at both dilutions and was chosen for further PCR analysis and probe design (see results section).

### Larval water preparation

We used laboratory-reared second instar *An. gambiae* s.s. larvae (G3 strain). The larvae were reared in plastic trays (20 × 18 × 7 cm) under controlled insectary conditions of temperatures 26–28 °C, relative humidity 70–80 % and 12:12 hour light: dark cycle and fed once daily on finely-ground TetraMin® fish food.

In a first experiment, we tested 12 different conditions by adding 50 mL of distilled, autoclaved water to 12 sterile 50 mL falcon tubes. We performed three biological replicates with three different larvae densities: 1, 3 and 6 larvae. One tube from each of the three densities was then sampled simultaneously at intervals of 1, 6, and 24 hours. Three negative control experimental habitats with no larvae were run in parallel for each condition.

In the second experiment, we added 1 L of distilled, autoclaved water to five 1 L glass bottles. This was followed by adding 2, 5, 10 and 20 larvae into each bottle with no larvae in the control bottle and left them standing in the enclosed environment of the PCR workstation for 24 hours and sampled three biological replicates from each condition.

For each replicate, larvae were first rinsed with distilled water to reduce chances of any carry over of eDNA from rearing tray water into the experimental habitats. All replicates were set up in a PCR workstation in a room separate from the main molecular laboratory to avoid contamination from aerial mosquito DNA.

### Extraction of eDNA from water samples

eDNA from the water samples was concentrated following a precipitation method ( Ficetola *et al.*, 2008). We sampled 15 mL of water into a sterile 50 mL falcon tube and immediately added 1.5 mL of 3 M sodium acetate solution followed by 11 mL of absolute ethanol and stored overnight at -20 °C. Samples were then centrifuged at 5000 rpm at 6°C for 1 hour. The supernatant was discarded, and the pellet retained for eDNA extraction. eDNA was extracted using ChargeSwitch® Forensic DNA Purification Kit, Invitrogen which is a magnetic bead-based system that isolates DNA based on changes in pH of the surrounding extraction buffers. The eDNA extraction followed manufactures instructions with some modifications including an overnight incubation at 4°C with 1 mL of lysis buffer, and 10 μL of Proteinase K, a lysis step at 56°C for 90 min., and samples eluted with 60 μL of the proprietary elution buffer.

### Quantitative PCR assays

In addition, novel *cyt b* TaqMan primer-probes that would distinguish the *An. gambiae* complex mosquitos from other species was designed and optimized for the primer pair 1 above. The primer probes had product length of 150 bp and on position 1114 at the 3’ end of the gene; double dye FAM labelled probes 5’- CCCACCCTTTAATTAGAATCGCTAA-3’ and 5’- CGGCATAGTGTATAGCTAGGAATAAT-3’ (PrimerDesign, UK). The probes were blasted against all known sequences in the NCBI database to confirm their specificity to *An. gambiae* s.l. complex mosquito species.

To detect eDNA from *An. gambiae* s.s. and *An. arabiensis* we used an existing TaqMan quantitative PCR protocol with probes targeting species-specific polymorphisms in the ribosomal DNA from the 3' 28S to 5' intergenic spacer region ( *IGS*) of the genome ( Walker *et al.*, 2007); hereafter ‘ *IGS* TaqMan’.

The qPCR standard curves for the two assays were performed by conducting a fivefold dilution series of genomic DNA from (a) *An. gambiae* s.s., (b) *An. arabiensis* and (c) a mixture from both species. Prior to this, Quant-iT™ PicoGreen® dsDNA Assay Kit (ThermoFisher) was used to determine the DNA concentration of the starting template for each series. From these standard curves, we aimed to determine: 1) linearity (on a log scale) of the qPCR across DNA concentrations; 2) detection limits for the assay; 3) quantitation equations for DNA present in an unknown sample.

For the experimental aquatic habitats, due to limiting reagents and time, we only conducted qPCR analysis on eDNA samples from 2, 5, 10, and 20 larvae in 1 L habitats. To test the limit of detection of the two assays, we conducted a fivefold dilution (1/5, 1/25 and 1/125) and ran *IGS* and *cyt b* assays for each with four technical replicates for each condition. Samples used in determining the standard curves and two no-template controls were included in each qPCR plate for the 1L habitats eDNA samples. The unknown eDNA samples were scored as positive if their Ct value fell within same range as the standard curve samples.

The total qPCR reaction volume was 20 μL and consisted of 1 μL primer probe pair (PrimerDesign, UK), 10 μL TaqMan® Gene Expression Master Mix (Applied Biosystems, USA), 4.5 μL nuclease free water and 4.5 μL eDNA template. The samples were then run on an Agilent Mx3005P qPCR System using the thermal profile: 95 °C for 10 minutes followed by 50 cycles of 92 °C for 15 seconds and 57 °C for 60 seconds. In the *IGS* assay, fluorescence was recorded through the FAM and VIC channels, with the FAM dye indicating *An. Arabiensis* detection and VIC indicating *An. gambiae* s.s. detection. In the *cyt b* assay, fluorescence was recorded through the FAM channel, and indicated the presence of *An. gambiae* s.l.

## Results

### Initial testing and validation

The eDNA PCR primers were designed as an initial step to test the extraction method and to ensure that the biological negative controls gave negative results. Both eDNA primers showed 100% amplification on control gDNA from *An. gambiae* s.s. (N=4 samples) at 1:10 concentration. At 1:100 dilutions, primer pairs 1 and 2 showed 100% and 75% amplification respectively with zero amplification in the negative controls. Based on this clear difference in performance and time convenience, primer pair 1 was chosen for subsequent PCR analysis and qPCR probe design. Sequencing results from the PCR positive samples in the two dilutions revealed that the primers were only binding to *An. gambiae* s.s *. cyt b* gene. The ChargeSwitch-extracted DNA showed 100% (N=8) amplification with the An. *gambiae* s.l. primer pair 1 with all negative controls giving showing no band.

A total of 74% replicates in which larvae were maintained in 50 mL of water (0.02, 0.06, and 0.12 larvae/mL) produced PCR bands. 86% of negative results were in replicates with a single larva (0.02 larvae/mL) that required at least 6 hours for the first positive result (1/3 replicates) and presented inconsistent detection at 24 hours (2/3 replicates; Table 1). All 12 replicates where 1 L of water was used (0.002 to 0.02 larvae/mL) were positive with stronger PCR bands than replicates with the same larval density in 50 mL for 24 h (e.g. 20 larvae in 1L vs. 1 larva in 50 mL). Moreover, replicates of the lowest larvae density in 1 L (2 larvae, 0.002 larvae/mL) gave a more consistent positive eDNA signal for 24 h than replicates with higher density (×10) in 50 mL (0.02 larvae/mL). The comparison between replicates of 1 L *vs.* 50 mL indicates a potential stochastic effect for eDNA detection giving more importance to the number of larvae than density for eDNA detection in small portion of water. All the four biological negative controls (with zero larvae) for each test condition showed no bands in the PCR.

**Table 1.** PCR detection of eDNA samples from containers with different volumes of water, numbers of larvae and occupation times.

No. of larvae	Vol. of water	Larvae density (larvae/mL)	Hours	PCR results	Band strength
1	50 mL	0.02	1	0/3	No band
3	50 mL	0.06	1	3/3	Weak
6	50 mL	0.12	1	3/3	Weak
0	50 mL	0	1	0/1	No band
1	50 mL	0.02	6	1/3	Weak
3	50 mL	0.06	6	3/3	Weak
6	50 mL	0.12	6	2/3	Weak
0	50 mL	0	6	0/1	No band
1	50 mL	0.02	24	2/3	Weak
3	50 mL	0.06	24	3/3	Weak
6	50 mL	0.12	24	3/3	Mid
0	50 mL	0	24	0/1	No band
2	1 L	0.002	24	3/3	Weak
5	1 L	0.005	24	3/3	Weak
10	1 L	0.01	24	3/3	Mid
20	1 L	0.02	24	3/3	Strong
0	1 L	0	24	0/1	No band

### IGS and cyt b qPCR Standard curves

**Figure 1.**
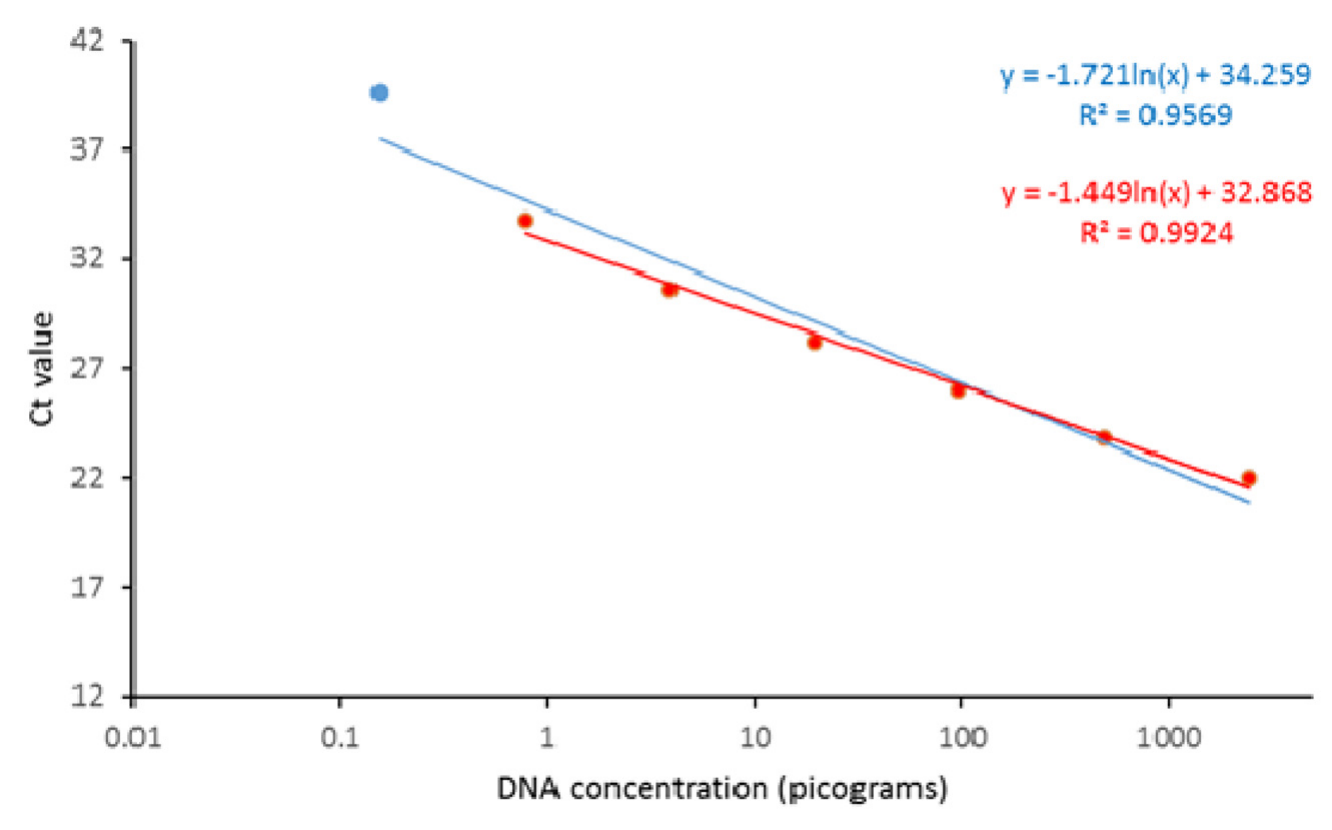
*Cyt b* TaqMan standard curve for across a fivefold dilution series of gDNA from *An. gambiae* s.s. The blue line shows a regression line fitted through all points, with associated equation in blue font (upper equation). The red line shows a line plotted through points excluding the lowest concentration sample, with associated equation in red font (lower equation).

The *cyt b* standard curve exhibited linearity in regression down to 0.776 picograms (pg) followed by a visually outlying quantification at the lowest dilution, 0.156 pg, Ct 39.65 ( Figure 1). The equation used for calculating concentration of downstream samples therefore excluded this lowest dilution ( Figure 1, red equation). From the fitted curve, the lower limit of detection was therefore taken as 0.776 pg hence any quantification below this might be downward-biased.

**Figure 2.**
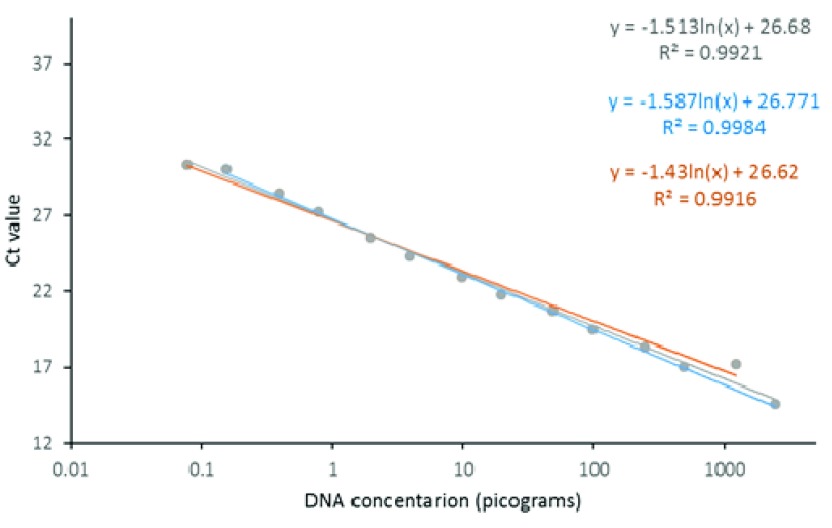
*IGS* TaqMan standard curve for across a fivefold dilution series of gDNA from *An. gambiae* s.s (blue line and equation), *An. arabiensis* (orange line and equation) and both (grey line and equation).

The *IGS* TaqMan exhibited a linear association between Ct values and DNA concentration within the concentration range tested. Standard curves for the probe targeting each species (i.e. *An. gambiae* s.s. and *An. arabiensis*) showed close similarity, which allowed data to be combined in a single model to produce a predictive equation, with more data points, for DNA detection from subsequent samples ( Figure 2). The *IGS* assay readily detected DNA at the lowest dilution of 0.156 pg and quantitation also appeared accurate throughout the range of dilutions.

### qPCR detection of larval presence from eDNA

Both the *IGS* and the *cyt b* TaqMan assays could detect *An. gambiae* s.s. eDNA in the undiluted samples of 2, 5, 10 and 20 larvae ( Table 2). All dilutions with 2 larvae (0.4 - 0.016 larvae/L) were negative, while all dilutions with 20 larvae were positive (0.2 - 4 larvae/L). For 10 larvae, both assays identified the second and third dilutions (0.08 - 0.4 larvae/L) with low consistency (~1/4 positive). Moreover, *IGS* TaqMan presented a better performance with 5 larvae, with positive detection (2/4) in the third dilution (0.04 larvae/L), than *cyt b* assay (only detected d1, 1 larvae/L).

**Table 2.** Comparison of results from *IGS* and *cyt b* TaqMan assays showing the number of positive samples in each of the four replicates and the average concentrations (pg) for each sample. The larval density was a fivefold dilution series (d1,2,3) for the initial number of larval in water sample, C. Dilutions whose eDNA concentration was not determined are noted as n.d. Minimum sample dilutions at which eDNA was detected are highlighted in bold.

2 larvae						5 larvae					
		Density	Positives	Av Conc	Std Dev			Density	Positives	Av Conc	Std Dev
**IGS**	C	**2**	**2/4**	0.0002	0.0002	**IGS**	C	5	4/4	0.006	0.002
	d1	0.4	0/4	n.d	n.d		d1	1	3/4	0.003	0.002
	d2	0.08	0/4	n.d	n.d		d2	0.2	0/4	n.d	n.d
	d3	0.016	0/4	n.d	n.d		d3	**0.04**	**2/4**	0.003	0.008
	NTC	0	0/2	n.d	n.d		NTC	0	0/2	n.d	n.d
***cyt b***	C	**2**	**2/4**	0.0306	0.0422	***cyt b***	C	5	4/4	0.0885	0.0244
	d1	0.4	0/4	n.d	n.d		d1	**1**	**1/4**	0.05101	0
	d2	0.08	0/4	n.d	n.d		d2	0.2	0/4	n.d	n.d
	d3	0.016	0/4	n.d	n.d		d3	0.04	0/4	n.d	n.d
	NTC	0	0/2	0	0		NTC	0	0/2	0	0
10 larvae						20 larvae					
		Density	Positives	Av Conc	Std Dev			Density	Positives	Av Conc	Std Dev
**IGS**	C	10	4/4	0.0078	0.0035	**IGS**	C	20	4/4	9.0527	1.0964
	d1	2	4/4	0.0017	0.0010		d1	4	4/4	1.7046	0.0821
	d2	0.4	2/4	0.0004	0.0002		d2	0.8	4/4	0.3758	0.0884
	d3	**0.08**	**1/4**	0.0002	0		d3	**0.16**	**4/4**	0.0818	0.0182
	NTC	0	0/2	0	0		NTC	0	0/2	0	0
***cyt b***	C	10	4/4	0.7848	0.4672	***cyt b***	C	20	4/4	27.2678	5.5316
	d1	2	3/4	0.2078	0.1813		d1	4	4/4	6.8140	1.8393
	d2	0.4	1/4	0.1270	0*		d2	0.8	4/4	1.7338	0.2231
	d3	**0.08**	**1/4**	0.0346	0		d3	**0.16**	**4/4**	0.3709	0.1233
	NTC	0	0/2	0	0		NTC	0	0/2	0	0

There was a correlation between larval densities and eDNA detection (38 (59%) positive samples for *IGS* and 32 (50%) for *cyt b -* Table 2) in both assays: *IGS*, *r* = 0.503, p = 0.047; and *cyt b, r* = 0.558, p = 0.025. However, there was also stochasticity in eDNA detection across the samples which reflected larval number in addition to larval concentration ( Table 2). For example, 0.2/L was readily detected from an initial sample containing 20 larvae but not in one originating from 5 larvae. Similarly, 0.08/L was undetectable in water originating from a sample with 2 larvae but was detectable in majority of replicates from the 10 larvae samples. This experiment suggested that depending on starting larval numbers, density of up to 0.04 larvae/L could be detectable.

## Discussion

Our study demonstrates that eDNA analysis using *IGS* and *cyt b* qPCR assays can be used to detect presence/absence and quantify *An. gambiae* s.l *.* mosquito larvae in a controlled laboratory experiment. The slightly better performance by the *IGS* assay suggests that the intergenic spacer region is a suitable target for eDNA primer/probe design. By virtue of the species specificity of polymorphisms in this genomic region, the *IGS* assay has the advantage to distinguish between *An. gambiae* s.s. and *An. arabiensis,* which may allow individually monitoring these primary malaria vectors by eDNA analysis. Assays targeting this region have the potential to distinguish among key species in other species complexes, which can be difficult using mtDNA.

We observed stochasticity in eDNA detection rates, using both PCR and qPCR, across the dilutions in the 50 mL and 1 L laboratory experimental habitats reflecting larval numbers in addition to density. For example, using PCR, replicates of the lowest larval density in 1 L (2 larvae, 0.002 larvae/mL) yielded a more consistent positive eDNA signal for 24 h than replicates with higher density (x10) in 50 mL (0.02 larvae/mL). Similarly, using qPCR, 0.4 larvae/L could be detected in the 10 larvae habitats but not in the 2 larvae habitats. Accurate detection and quantification in natural habitats may depend of a minimum number of larvae without a direct correlation with density. This could translate to organisms not being detected when they are present in small numbers ( Ficetola *et al.*, 2008). Such stochasticity can be reduced through replication by either using multiple environmental samples collected at different points around the habitat for pooled DNA extraction or multiple amplification of the extracted DNA ( Jerde *et al.*, 2011; Thomsen & Willerslev, 2015) though this will increase costs and risk of false positives. Time of occupancy as well as biomass of the target species influences eDNA concentration and detection probability.

Based on this laboratory experiment, we extrapolate that mosquito surveillance by eDNA analysis would be better for detecting larvae in habitats with many larvae at low densities than in aquatic habitats with few larvae and higher densities. Since our quantifications were based on a dilution series from an initial larval density that could be higher than those found in natural mosquito habitats, it is not clear how these results can be extrapolated to quantify mosquito biomass in natural breeding habitats necessitating further evaluations on field applicability.

This study has some limitations. First with the dilution series used for our standard curves in which the lowest limit of eDNA detection was high making any quantification below this limit to be extrapolated from the standard curve equation which could potential introduce a bias. Secondly, we used *An. gambiae* s.s. and *An. arabiensis* gDNA for the standard curves instead of eDNA. Thirdly, the laboratory habitat experiments were only conducted using *An. gambiae* s.s. larvae. Since *An. gambiae* s.s. and *An. arabiensis* occur sympatrically in natural habitats ( Chen *et al.*, 2008), further validations should include experiments where the two species are occupying the same habitat. However, this did not affect our overall conclusion as a qPCR result from DNA mixture of the two-species had a similar linear relationship as for each on its own.

Further studies need to be done to determine the applicability of eDNA analysis on detecting larvae belonging to the *An. gambiae* complex under field conditions. Evaluation of this tool in monitoring malaria vector species which are difficult to detect in breeding sites, such as *An. funestus* ( Gimnig *et al.*, 2001), should also be carried out especially in habitats where traditional sampling methods are logistically difficult such as rice fields.

## Conclusion

We have successfully detected and quantified *An. gambiae* s.s. mosquito larvae using eDNA from water samples. The stochasticity observed in eDNA detection suggest that mosquito surveillance by eDNA analysis would be better for detecting larvae in habitats with many larvae at low densities than in aquatic habitats with few larvae at higher densities. The *IGS* assay previously designed for identifying wild-type *An. gambiae* s.s. and *An. arabiensis* can distinguish aquatic stages of these primary malaria vectors using extracellular DNA extracted from water collected in the breeding habitats. This is important as eDNA detection to species level rather species complex level is achievable which could potentially save costs and time employed in monitoring these primary malaria vectors.

## Data Availability

Dataset available from OSF: http://doi.org/10.17605/OSF.IO/FRH28 ( Odero *et al.,* 2018)

Data are available under the terms of the Creative Commons Zero "No rights reserved" data waiver (CC0 1.0 Public domain dedication).
