# Supplementary material for: Detection and quantification of Anopheles gambiae sensu lato mosquito larvae in experimental aquatic habitats using environmental DNA (eDNA)
Source: Wellcome Open Res. 2018 Mar 8;3:26. [Version 1] doi: 10.12688/wellcomeopenres.14193.1 (PMC5981191; doi:10.12688/wellcomeopenres.14193.1)
Supplement: Peer review history

# Peer review: Referee response for version 1

**DOI:** 10.21956/wellcomeopenres.15439.r33150

**Published:** 2018-06-06

**Authors:** Iliana Bista

**Affiliations:** 1 Wellcome Trust Sanger Institute, Hinxton, UK

Odero and co-authors use eDNA analysis to detect *Anopheles gambie *in laboratory environment, through the use of direct PCR and qPCR methodology. They target IGS and cyt b regions, at different timepoints and varying larvae densities. The work demonstrates positive detection of species in laboratory conditions. This is a good proof of concept study for PCR based detection of *A. gambiae*, with interesting possibilities for field application, since improving detection of *A. gambiae*, which is an important malaria vector, could be beneficial for mosquito management in afflicted areas.

 The paper is generally well written, with good use of language and mostly clear descriptions of methodology and discussion. A small number of unclear points exist in methodology, as well as interpretation of results, and final conclusions. More detailed comments are provided below.

  

1. In Methods section “Larval water preparation”, please clarify the description of conditions and biological replicates. It is my understanding that the 50ml set-up was replicated 3 times, while the 1L bottle experiment was based on a single bottle per larva number (no biological replicates). Yet, at the end of 3rd paragraph it says “…sampled three biological replicates for each condition”. This doesn’t apply to the second experiment. Collection of multiple samples/PCRs per treatment constitutes technical, not biological replicates.
2. In methods section: “All replicates were set up in a PCR workstation in a room separate from the main molecular laboratory to avoid contamination from aerial mosquito DNA. “. eDNA detection is sensitive to contamination, especially through amplified DNA products. Was amplified mosquito DNA handled previously in that PCR workstation? Potential contamination from PCR products could occur by setting up the experiment in that space. The good thing is that the negative controls appear negative in all cases, but there was only one technical replicate for the negatives compared to 3 for other treatments. Please clarify why only one PCR was run for negative controls.
3. The authors refer to calculated larvae density throughout the text. Please explain how was the size of the larvae biomass calculated in order to estimate larval density. Were the larvae weighed or measured prior to being placed in the experimental set-up?
4. At the end of Introduction, beginning of Discussion, and in Conclusions, the authors claim to have established an assay “…capable of simultaneous differentiation of the two key East African malaria vectors *An. gambiae s.s*. and *An. arabiensis*.”. Nevertheless, no direct comparison of eDNA from the two species is performed in experiments. The capability of the IGS region to discriminate between the two species, if already documented in the literature, could provide an advantage for use of this assay in the field. But the experiments conducted herein don’t directly prove this claim. It would be more appropriate to provide this as a suggestion rather than a finding. Instead, the authors could augment the Discussion section by referencing and discussing related literature which supports the ability of the IGS region to make species specific detection.
5. As a general note, conducting such experiments in laboratory environment, using sterile water, is probably far from sampling a natural environment and hard to generalize. Suggestions for future work should include testing this assay in field conditions or even mesocosms. A number of environmental factors can influence detection, by affecting degradation rates, or interfering with PCR, which should be taken into consideration for field applications.
6. The ability of eDNA based methodologies to effectively reflect biomass/ abundance is still a controversial issue in the eDNA field, with the majority of the literature reporting inaccurate detection, mostly related to PCR bias. On the other hand, presence/absence detection is more accurate and reliable, especially when technical replicates are used. Consider rephrasing text in relation to accuracy of eDNA based quantification of species detections.
7.  Consider enhancing the discussion section by incorporating related literature.

 

**Minor points: **

- Table 2 legend – “larval in water” change to “larvae in water”.

 

- “For the experimental aquatic habitats, due to limiting reagents”, change to limited.
- “Time of occupancy as well as biomass of the target species influences eDNA concentration   and detection probability.” Please provide reference.

I have read this submission. I believe that I have an appropriate level of expertise to confirm that it is of an acceptable scientific standard.

# Peer review: Referee response for version 1

**DOI:** 10.21956/wellcomeopenres.15439.r33151

**Published:** 2018-05-30

**Authors:** Mathew Seymour

**Affiliations:** 1 School of Biological Sciences, Molecular Ecology and Fisheries Genetics Laboratory (MEFGL), Bangor University, Bangor, UK

Odero *et al* presents a clearly written study using eDNA based methods to detect mosquito larvae, which as the authors indicate could be a highly relevant application. The study and experiment seem in order, but some clarity on the specificity of the primers and comparison with existing eDNA based biomonitoring programs would be advisable. Additionally, the conclusion are a bit confusing at present, particularly regarding the link between species detection and density derived from the experiment.

 Introductions

 1) Please provide an explanation for larval source management (LSM). The method is not common knowledge, at least for readers such as myself.

 2) Quantifying numbers of individuals/biomass using eDNA is still being validated in the field. The authors cite Thomsen and Willerslev 2015 ^1^, which is a review article. Studies that have assessed the relationship between eDNA and community numbers include Doi, H. *et al. *(2015) ^1^ and Iliana, B. *et al. *(2018) ^3^.

 3) There are other key factors influencing eDNA degradation that could be mentioned. Environmental DNA degradation dynamics are also influenced by pH as shown in semi-natural and laboratory conditions

 Seymour, M. *et al. *(2018) ^4^ and Strickler, K. M *et al.* (2015) ^5^.

 4) The effects of natural sunlight on eDNA degradation is less supported as there are also studies that suggest natural UV levels have no effect on eDNA degradation

 Mächler, E et al. (2018) ^6^ and Andruszkiewicz, E. A et al. (2017) ^7^.

 5) Please also consider that sedimentation will also influence detection of eDNA ^8^.

 Methods

 6) Please consider including all relevant MIQE information (could be an appendix), which will help with promoting the application of the proposed primers for eDNA based approaches as suggested in Goldberg et al. 2016 ^9^.

 Results

 7) Sections of the results are interpretative and should be moved to the discussion or rewritten.

 “The comparison between replicates of 1 L vs. 50 mL indicates a potential stochastic effect for eDNA detection giving more importance to the number of larvae than density for eDNA detection in small portion of water. All the four biological negative controls (with zero larvae) for each test condition showed no bands in the PCR.”

 “However, there was also stochasticity in eDNA detection across the samples which reflected larval number in addition to larval concentration (Table 2). For example, 0.2/L was readily detected from an initial sample containing 20 larvae but not in one originating from 5 larvae. Similarly, 0.08/L was undetectable in water originating from a sample with 2 larvae but was detectable in majority of replicates from the 10 larvae samples. This experiment suggested that depending on starting larval numbers, density of up to 0.04 larvae/L could be detectable.”

 8) Table 2. Please consider rewording ‘density’ to ‘dilution’ as ‘density’ might imply number of larvae.

 9) Consider rewriting the opening to the second paragraph of the discussion to clarify the main point. My understanding is that lower concentrations/densities increased stochasticity in detection rates, which is expected based on other studies that use similar methods for biomonitoring programs

 Biggs, J. *et al. *(2015) ^10^ and Jerde, C. L. *et al.* (2013) ^11^.

 10) Please consider comparing the findings with existing eDNA based biomonitoring programs with the findings presented here. It is a bit of an elephant in the room when reading the discussion.

 11) Were in silico analyses performed to validate the specificity of the primers? Lab based specificity test would be the next step before field applications, but can anything be said about the initial primer selection procedures based on the blast assessment?

 12) Could this sentence be rephrased please? In the context of the previous sentence, it is unclear how the similarity in the linear relationships helps validate the specificity of the primers.

 13) “However, this did not affect our overall conclusion as a qPCRresult from DNA mixture of the two-species had a similar linear relationship as for each on its own.”

 The second sentence in the conclusion is confusing. Could the statement be rewritten to simply state that eDNA based detection is less stochastic when sampling from higher density populations?

 14) Please specify what the ‘this’ in the last sentence is referring to.

I have read this submission. I believe that I have an appropriate level of expertise to confirm that it is of an acceptable scientific standard, however I have significant reservations, as outlined above.

## References

[1] ThomsenPWillerslevE: Environmental DNA – An emerging tool in conservation for monitoring past and present biodiversity. *Biological Conservation*.2015;183: 10.1016/j.biocon.2014.11.019 4-18 10.1016/j.biocon.2014.11.019

[2] DoiHUchiiKTakaharaTMatsuhashiSYamanakaHMinamotoT: Use of droplet digital PCR for estimation of fish abundance and biomass in environmental DNA surveys. *PLoS One*.2015;10(3) : 10.1371/journal.pone.0122763 e0122763 10.1371/journal.pone.0122763 25799582PMC4370432

[3] BistaICarvalhoGRTangMWalshKZhouXHajibabaeiMShokrallaSSeymourMBradleyDLiuSChristmasMCreerS: Performance of amplicon and shotgun sequencing for accurate biomass estimation in invertebrate community samples. *Mol Ecol Resour*.2018; 10.1111/1755-0998.12888 10.1111/1755-0998.12888 29667329

[4] SeymourMDuranceICosbyBRansom-JonesEDeinerKOrmerodSColbourneJWilgarGCarvalhoGde BruynMEdwardsFEmmettBBikHCreerS: Acidity promotes degradation of multi-species environmental DNA in lotic mesocosms. *Communications Biology*.2018;1(1) : 10.1038/s42003-017-0005-3 10.1038/s42003-017-0005-3 PMC612378630271891

[5] StricklerKFremierAGoldbergC: Quantifying effects of UV-B, temperature, and pH on eDNA degradation in aquatic microcosms. *Biological Conservation*.2015;183: 10.1016/j.biocon.2014.11.038 85-92 10.1016/j.biocon.2014.11.038

[6] MächlerEOsathanunkulMAltermattF: Shedding light on eDNA: neither natural levels of UV radiation nor the presence of a filter feeder affect eDNA-based detection of aquatic organisms. *PLoS One*.2018;13(4) : 10.1371/journal.pone.0195529 e0195529 10.1371/journal.pone.0195529 29624607PMC5889167

[7] AndruszkiewiczEASassoubreLMBoehmAB: Persistence of marine fish environmental DNA and the influence of sunlight. *PLoS One*.2017;12(9) : 10.1371/journal.pone.0185043 e0185043 10.1371/journal.pone.0185043 28915253PMC5600408

[8] JerdeCOldsBShogrenAAndruszkiewiczEMahonABolsterDTankJ: Influence of Stream Bottom Substrate on Retention and Transport of Vertebrate Environmental DNA. *Environmental Science & Technology*.2016;50(16) : 10.1021/acs.est.6b01761 8770-8779 10.1021/acs.est.6b01761 27409250

[9] GoldbergCTurnerCDeinerKKlymusKThomsenPMurphyMSpearSMcKeeAOyler-McCanceSCornmanRLaramieMMahonALanceRPilliodDStricklerKWaitsLFremierATakaharaTHerderJTaberletP: Critical considerations for the application of environmental DNA methods to detect aquatic species. *Methods in Ecology and Evolution*.2016;7(11) : 10.1111/2041-210X.12595 1299-1307 10.1111/2041-210X.12595

[10] BiggsJEwaldNValentiniAGaboriaudCDejeanTGriffithsRFosterJWilkinsonJArnellABrothertonPWilliamsPDunnF: Using eDNA to develop a national citizen science-based monitoring programme for the great crested newt (Triturus cristatus). *Biological Conservation*.2015;183: 10.1016/j.biocon.2014.11.029 19-28 10.1016/j.biocon.2014.11.029

[11] JerdeCChaddertonWMahonARenshawMCorushJBudnyMMysorekarSLodgeD: Detection of Asian carp DNA as part of a Great Lakes basin-wide surveillance program. *Canadian Journal of Fisheries and Aquatic Sciences*.2013;70(4) : 10.1139/cjfas-2012-0478 522-526 10.1139/cjfas-2012-0478

# Peer review: Referee response for version 1

**DOI:** 10.21956/wellcomeopenres.15439.r31723

**Published:** 2018-04-20

**Authors:** Hideyuki Doi

**Affiliations:** 1 Graduate School of Simulation Studies, University of Hyogo, Kobe, Japan

I enjoyed reading the manuscript and found that the paper was technically sound. However, the qPCR methods was not according to MIQE checklist ^1^, e.g., R2 and PCR effciency of the standard curves, limited of detection, and others. Also, ethanol volume for precipitation method was quite small (11 ml), generally using 33 mL for 15 mL of sampled water.

I have read this submission. I believe that I have an appropriate level of expertise to confirm that it is of an acceptable scientific standard.

## References

[1] BustinSABenesVGarsonJAHellemansJHuggettJKubistaMMuellerRNolanTPfafflMWShipleyGLVandesompeleJWittwerCT: The MIQE guidelines: minimum information for publication of quantitative real-time PCR experiments. *Clin Chem*.2009;55(4) : 10.1373/clinchem.2008.112797 611-22 10.1373/clinchem.2008.112797 19246619

# Peer review: Referee response for version 1

**DOI:** 10.21956/wellcomeopenres.15439.r31725

**Published:** 2018-04-06

**Authors:** Stefano Mariani

**Affiliations:** 1 School of Environment and Life Sciences, University of Salford, Salford, UK

This is a clearly written work reporting on a clearly designed experiment.

 The authors provide novel molecular resources for the detection and quantification of trace DNA for two major malaria vectors.

 In general, the work is sound, but it would benefit from a couple of modifications and some downscaling of the conclusions.

 My major comments are:

 1) the authors extrapolate and conclude that the method devised will suit larger waterbodies with many larvae at low densities, rather than smaller environments with fewer larvae at possibly higher densities. I do not think the data support this conclusion. The data may perhaps suggest something along those lines, but mostly because the study focused on two different sets of artificial habitats, one smaller and one larger, but not fully comparable with one another (neither in terms of replication or stocking densities) and only loosely comparable with real habitats where Anopheles larvae can be found. I am not a mosquito expert, but I am familiar with the complexity of larger aquatic habitats, and stochasticity, patchiness, physico-chemical variability, microbiological activity, etc. will all introduce an array of complications, which may in fact yield a greater negative/misses in larger habitats than in smaller ones, if densities are low. I believe this is a major aspect that the authors need to mitigate and rearrange. The concluding statements should focus more on the efficiency of the molecular tools, their species-specific effectiveness, and their good quantitative performance.

 2) The authors never mention explicitly why it is so important to distinguish between the two species of Anopheles. I work on biodiversity conservation and not on infectious diseases, but I presume that the need to identify different species will vary depending on the key management need. At least a paragraph on this should be added.

 3) The authors explain quite clearly that targeting larval assemblages may be a more efficient way to control these vectors. However, they mention the "wider spectrum of pesticides" as an effective tool; but from an environmental conservation point of view, this sounds somewhat reckless. I appreciate that malaria is a huge problem in many parts of the world, but I would like to see some caveats about the indiscriminate use of pesticide in aquatic habitats, as their pernicious effects, on both ecosystems and human health, have been widely documented.

 I also list below a few minor points to be amended:

 - please remove the word "an" before "IGS" in the methods paragraph within the abstract.

 - in the methods, it would be nice to explain more clearly that the larvae that were reared in the trays were then transferred to the falcon tubes, which then represented the actual "small experimental habitat".

 - I note that autoclaved/distilled water is always used in the experiments. This provides a medium that will depart remarkably from natural conditions. When extrapolating, the authors should caution about this, for the benefit of anyone trying to used these tools in a natural setting, where humic substances, chemical inhibitors, microbiological activity, etc. will place these molecular probes in a very challenging, unchartered territory...

 - In the results, it would be nice to reword the sentence mentioning the "86% of negative results..." changing it into "x out of y negative results..." because earlier there is another percentage and it may cause confusion.

 - also in the results, it isn't very clear how eDNA "detection" can be correlated with "density". Detection is a binary entity (presence/absence)... do the authors mean "concentration"?

I have read this submission. I believe that I have an appropriate level of expertise to confirm that it is of an acceptable scientific standard, however I have significant reservations, as outlined above.
